# Racial Disparities in Cytoreductive Surgery and Hyperthermic Intraperitoneal Chemotherapy: Does Aggressive Surgical Treatment Overcome Cancer Health Inequities?

**DOI:** 10.3389/fonc.2022.899488

**Published:** 2022-06-08

**Authors:** Devon C. Freudenberger, Xiaoyan Deng, Vignesh Vudatha, Andrea N. Riner, Kelly M. Herremans, Dipankar Bandyopadhyay, Leopoldo J. Fernandez, Jose G. Trevino

**Affiliations:** ^1^ Division of Surgical Oncology, Department of Surgery, Virginia Commonwealth University School of Medicine, Richmond, VA, United States; ^2^ Department of Biostatistics, Virginia Commonwealth University School of Medicine, Richmond, VA, United States; ^3^ Department of Surgery, University of Florida College of Medicine, Gainesville, FL, United States

**Keywords:** peritoneal carcinomatosis, cytoreductive surgery, HIPEC (heated intraperitoneal chemotherapy), surgical outcomes, racial disparities

## Abstract

**Background:**

Advanced cancer states perpetuate health-related disparities. Peritoneal-based cancers are clinically advanced cancers that present a significant clinical dilemma. Peritoneal cancers are managed aggressively with cytoreductive surgery (CRS) and hyperthermic intraperitoneal chemotherapy (HIPEC). While racial and ethnic disparities are prevalent in cancer, there are no studies investigating if racial disparities exist in patients with peritoneal carcinomatosis managed with CRS and HIPEC. We hypothesized that this advanced disease state further delineates racial disparities.

**Methods:**

A retrospective chart review was conducted on patients with peritoneal carcinomatosis receiving CRS and HIPEC at a single institution from January 1, 2017-October 4, 2021. Descriptive statistics were used to compare racial groups. The Cox Proportional Hazards Model and Log Rank Test were used for multivariate and overall survival analysis.

**Results:**

In total, 67 patients underwent CRS and HIPEC, of which 41 (61.2%) were White, 20 (29.8%) were Black, 3 (4.5%) were Asian, and 3 (4.5%) were Other race. When compared to White patients, Black patients had lower income (p=0.0011), higher incidence of hypertension (p=0.0231), and lower performance status (p=0.0441). Cancer type, including colorectal, appendiceal, ovarian, etc., was similar between groups (p=0.8703). Despite these differences in sociodemographic and morbidity factors, when comparing Black patients to White patients, there were no differences in peritoneal cancer index score (13.2 vs. 12.3, p=0.6932), estimated blood loss (748 vs. 655 mL, p=0.6332), minor/major complication rates (1.1 vs. 1.2, p=0.7281; 0.4 vs. 0.7, p=0.3470, respectively), 30-day readmission rates (25.0% vs. 17.1%, p=0.6210), disease recurrence (40.0% vs. 51.2%, p=0.3667), or 30-day mortality (0.0% vs. 2.4%, p=1.0000). Overall survival was similar for Black and White patients (p=0.2693). The occurrence of a major complication was the only factor associated with overall survival (HR 2.188 [1.502, 3.188], p< 0.0001).

**Conclusions:**

Despite differences in patient socioeconomic factors and comorbid conditions, outcomes were similar between Black and White patients receiving CRS and HIPEC at our institution. While larger studies with more diverse patient populations are needed to confirm these findings, our data provide evidence that aggressive surgical management across diverse patient populations allows for equitable outcomes.

## Introduction

Advanced cancer states perpetuate health-related disparities through multiple mechanisms, including tumor biology, genetics, and sociodemographic factors (1). There has been much effort to examine and mitigate these disparities and provide more equitable care for diverse patient populations. Peritoneal carcinomatosis, however, is one such advanced cancer that has largely been overlooked in this realm of research.

Peritoneal carcinomatosis, or peritoneal surface malignancy/metastasis, is the dissemination of cancer along the peritoneum of the abdominal cavity. This peritoneal surface malignancy/metastasis occurs primarily as peritoneal mesothelioma or secondarily to a variety of abdominal and gynecologic cancers including colorectal, appendiceal, gastric, ovarian, and fallopian cancer (2) and represents a Stage IV cancer that is localized to the peritoneal lining. The presence of peritoneal carcinomatosis tends to yield a poor prognosis for patients and previous dogma viewed peritoneal carcinomatosis as an incurable systemic disease process. Fortunately, treatment advances have led to a paradigm shift where peritoneal carcinomatosis is viewed as a localized, potentially curable disease. This shift in clinical management has primarily occurred with the introduction of cytoreductive surgery (CRS) and hyperthermic intraperitoneal chemotherapy (HIPEC), which have been shown to improve survival in select patients (3).

CRS and HIPEC is an advanced, complex, morbid, and aggressive surgical treatment modality for peritoneal surface malignancies. During this treatment modality all macroscopic resectable tumor is removed from the abdomen, which can include a variety of anatomic resections specific to the patient, such as peritonectomy, enterectomy, colectomy, cholecystectomy, omentectomy, hysterectomy, etc. Heated chemotherapy specific to the cancer, is then infused into the abdomen to assist in destruction of any remaining tumor deposits and microscopic disease (4). Morbidity and mortality are high (5), but CRS and HIPEC has been found to have lower 30-day morbidity and mortality than other complex surgical procedures such as an esophagectomy, pancreaticoduodenectomy, and hepatectomy (6). Given the known risks of morbidity and mortality associated with this procedure, it is imperative to identify patient populations that might not only be disproportionately affected by this disease but also those that will clinically benefit from such aggressive surgery.

Few studies have investigated the disparities present in patients with peritoneal carcinomatosis treated with CRS and HIPEC. The limited number of currently published studies have focused primarily on the impact of socioeconomic status and insurance status on patient outcomes, overlooking possible racial disparities within this patient population (7–9). Racial disparities exist in the diagnosis, treatment, management, and survival of cancer (10–15). Black patients have been shown to have higher rates of cancer-related mortality when compared to White patients (11, 16). Additionally, racial disparities are further perpetuated in the surgical treatment of cancer. Black patients undergoing major cancer surgery have been shown to have worse postoperative outcomes, including more complications, higher rates of in-hospital mortality, higher likelihood of needing postoperative blood transfusions, and longer hospital stays (17, 18). Recent encouraging data has shown that cancer surgery-related mortality has improved for both Black and White patients, but Black patients continue to be disproportionately affected compared to White patients (14). Therefore, we feel it is imperative that cancer-related investigations on higher risk and reward surgery in aggressive disease processes for racial/ethnic diverse patient populations should be investigated.

To our knowledge, there have been no studies investigating potential racial disparities in perioperative and postoperative outcomes for patients receiving CRS and HIPEC in the setting of peritoneal carcinomatosis. The aim of this study was to investigate this amongst patients receiving CRS and HIPEC at a single high-volume, tertiary institution. We hypothesized that racial disparities exist amongst patients receiving CRS and HIPEC, and thus, should be identified for improvement of patient outcomes and equity of care.

## Materials and Methods

### Patient Selection and Data

A retrospective chart review was completed for all patients who underwent CRS and HIPEC at our institution from January 1, 2017 to October 4, 2021. Patients eligible for inclusion in this study were 18 years of age and older and received first-time CRS and HIPEC for peritoneal carcinomatosis. Patients were excluded if they were less than 18 years old or received HIPEC exclusively for palliation of ascites. This study was approved by the institutional review board at Virginia Commonwealth University Health System.

Clinical data including patient demographics, risk factors, oncologic history, and intraoperative and postoperative outcomes were obtained from the electronic medical record for each patient. Demographics included age, sex, race (White, Black, Asian, or Other), ethnicity (Hispanic or Non-Hispanic), insurance status (private payor or government-based payor), distance traveled to the hospital (obtained by calculating the distance from the patient’s listed zip code city center to the treating medical center), and median household income (obtained using the patient’s listed zip code and US Census Data from the American Community Survey 5-year estimates from 2015 to 2019 (19)).

Risk factors included the patient’s preoperative American Association of Anesthesiologist Physical Status Classification System score (ASA score) and Eastern Cooperative Oncology Group Performance Status (ECOG-PS), and presence of comorbidities including hypertension, diabetes, chronic obstructive pulmonary disease (COPD), coronary artery disease, chronic kidney disease, and current smoking status. Oncologic history included the patient’s type of cancer and receipt of neoadjuvant chemotherapy prior to CRS and HIPEC.

Intraoperative variables included the length of surgery, calculated peritoneal cancer index (PCI), cytoreduction score (CC score), intraoperative receipt of blood transfusion, estimated blood loss (EBL), number of bowel anastomoses created, and creation of an ostomy. Postoperative variables included minor complications defined by the Clavien-Dindo classification types I-II and major complications defined by the Clavien-Dindo classification types III-IV within 30 days of surgery, length of hospital stay, readmission within 30 days of surgery, 30-day mortality, postoperative recurrence of disease defined by radiographic or biopsy-proven evidence, length of follow-up, and length of survival. Length of survival was calculated from the date of surgery to the patient’s known date of death or date of last record in the institution’s electronic health system.

### Statistical Analysis

The data were stratified by racial groups. Differences between racial groups’ demographic factors, preoperative risk factors, intraoperative outcomes, and postoperative outcomes were compared using descriptive and inferential statistics.

Multivariate analysis for clinical factors associated with survival was performed with the Cox Proportional Hazards Model. Explanatory factors included in the model were age, sex, race, insurance, income, neoadjuvant chemotherapy, minor complications, major complications, readmission at 30 days, and recurrence of cancer. The backward selection method was used. The significance level for removing effects from the model was specified at 0.1. Overall survival, the primary outcome, was also calculated and the Log Rank Test was used to compare the distribution of survival time between Black and White patients. Lastly, a power analysis was performed using a two-sided Log Rank Test to ensure adequate sample size for detecting differences in results. An alpha-value of 0.05 was used for determining significance. All statistical analyses were completed using SAS Version 9.4 (Cary, NC).

## Results

### Patient Characteristics

A total of 67 patients underwent CRS and HIPEC for peritoneal carcinomatosis during the specified time period and met inclusion criteria. The racial breakdown included 41 (61.1%) White patients, 20 (29.9%) Black patients, 3 (4.5%) Asian patients, and 3 (4.5%) patients listed as Other race. Given the small sample sizes for Asian and Other races, these patients were excluded from further analysis.

Patient demographics, preoperative risk factors, and oncologic history are presented in **Table 1**. Age, distribution of sex, and preoperative body mass index (BMI) were similar between Black and White patients. In terms of preexisting comorbidities, Black patients had higher rates of hypertension requiring medication compared to White patients (70.0% vs. 39.0%, p=0.0231), but otherwise comorbidities were present in both populations at comparable rates. Preoperative assessment of risk according to the ASA Score was similar between groups (p=0.9795). However, preoperative patient performance status as measured *via* the ECOG-PS was worse in Black patients compared to White patients (p=0.0441).

**Table 1:** Patient demographics and preoperative demographics by race.

	Black (n = 20)	White (n = 41)	*p*-value
Age (years)	55.5 (36.0-73.0)	55.3 (23.0-75.0)	0.9540
Sex			0.9416
Female	12 (60.0%)	25 (61.0%)	
Male	8 (40.0%)	16 (39.0%)	
BMI (kg/m^2^)	31.9 (21.8-53.9)	30.0 (18.4-57.8)	0.3483
Insurance			0.9354
Private	11 (55.0%)	23 (56.1%)	
Government	9 (45.0%)	18 (43.9%)	
Household Income (USD, mean)	53,719 (27,063-92,069)	69,294 (36,379-107,321)	0.0011
Distance Traveled (miles, mean)	34.9 (3.0-102.0)	63.0 (4.1-822.0)	0.3596
Comorbidities			
Hypertension	14 (70.0%)	16 (39.0%)	0.0231
Coronary artery disease	0 (0.0%)	5 (12.2%)	0.1620
Diabetes mellitus	4 (20.0%)	10 (24.4%)	0.7019
COPD	1 (5.0%)	1 (2.4%)	1.0000
Chronic kidney disease	1 (5.0%)	0 (0.0%)	0.3279
Current Smoker	2 (10.0%)	2 (4.9%)	0.5915
ASA Score			0.9795
1	0 (0.0%)	0 (0.0%)	
2	3 (15.0%)	7 (17.1%)	
3	16 (80.0%)	32 (78.1%)	
4	1 (5.0%)	2 (4.9%)	
5	0 (0.0%)	0 (0.0%)	
ECOG-PS			0.0441
0	11 (55.0%)	34 (82.9%)	
1	8 (40.0%)	7 (17.1%)	
2	1 (5.0%)	0 (0.0%)	
Primary Cancer Type			0.8703
Appendiceal	6 (30.0%)	10 (24.4%)	
Colorectal	7 (35.0%)	16 (39.0%)	
Esophageal	0 (0.0%)	1 (2.4%)	
Gastric	1 (5.0%)	1 (2.4%)	
Ovarian/Fallopian	2 (10.0%)	8 (19.5%)	
Small bowel	1 (5.0%)	2 (4.9%)	
Other	3 (15.0%)	3 (7.3%)	
Neoadjuvant Chemotherapy	13 (65.0%)	25 (61.0%)	0.7608
Preoperative Albumin	4.4 (3.1-4.8)	4.2 (3.6-5.0)	0.1701

Socioeconomic factors were analyzed as well to ascertain any differences in social determinants of health. Both groups were insured with private or government insurance at similar rates; no patient was uninsured. White and Black patients traveled similar distances to the medical center for treatment (63.0 vs. 34.9 miles, p=0.3596). Black patients had significantly lower household income than White patients ($53,719 vs. 69,294, p=0.0011).

With respect to patient oncologic history, the type of cancer as well as receipt of neoadjuvant chemotherapy were similar between racial groups (p=0.8703 and p=0.7608, respectively). In total, the most common cancers were of colorectal (37.8%) and appendiceal (26.2%) origin.

### Intraoperative and Postoperative Clinical Outcomes

Intraoperative and postoperative outcomes by race are presented in **Table 2**. A complete breakdown of each complication is reported in **Supplementary Table 1**. At the time of surgery, the mean PCI scores were similar between Black and White patients indicating similar extents of peritoneal disease (13.2 vs. 12.3, respectively). Length of surgery, estimated blood loss, the number of anastomoses created, and creation of an ostomy were similar between Black and White patients as well.

**Table 2:** Patient intraoperative and postoperative outcomes within 30 days of surgery by race.

	Black (n = 20)	White (n = 41)	*p*-value
PCI Score	13.2 (2-35)	12.3 (3-26)	0.6932
Length of Surgery (min)	590 (386-780)	642 (367-1098)	0.2975
EBL (mL)	748 (100-2500)	655 (50-3000)	0.6332
No. of anastomoses (median)	1 (0-3)	1 (0-3)	0.6290
Ostomy Creation	2 (10.0%)	5 (12.2%)	1.0000
Hospital LOS (days)	13.1 (5.0-26.0)	11.6 (3.0-48.0)	0.5012
Minor Complications	1.1 (0-3)	1.2 (0-5)	0.7281
Major Complications	0.4 (0-4)	0.7 (0-8)	0.3470
Total Complications	1.5 (0-7)	2.0 (0-11)	0.4579
Readmission within 30 days	5 (25.0%)	7 (17.1%)	0.6210
30-day mortality	0 (0.0%)	1 (2.4%)	1.0000
Recurrence after surgery	8 (40.0%)	21 (51.2%)	0.3667

Postoperatively, outcomes were similar between Black and White patients. The mean complication rates for both minor and major complications occurring within 30 days of surgery were similar between Black and White patients. Only one type of postoperative complication was noted to be statistically significant and higher in one group in relation to the other; Black patients experienced higher rates of prolonged intubation (defined as remaining intubated for greater than 48 hours after surgery) compared to White patients (15.0% vs. 0.0%, p=0.0317). Black and White patients had similar lengths of hospital stay (13.1 vs 11.6 days, respectively; p=0.5012). Readmission to the hospital within 30 days of discharge was also similar between races (p=0.6210). Recurrence of disease after surgery, as evidenced radiographically or biopsy-proven, occurred in 47.5% (29/61) of the patients, but recurrence rates were similar amongst Black and White patients (p=0.3677). Mortality within 30 days of the index operation was not statistically different between Black and White patients (0.0% vs. 2.4%, p=1.000).

### Multivariate and Survival Analysis

Multivariate analysis was conducted to identify factors associated with survival. After controlling for explanatory factors, only sex and the occurrence of major complication were included in the final model. The occurrence of a major complication was the only factor, however, associated with survival (HR: 2.188, 95% CI 1.502-3.188, p<0.001), indicating that for each major complication suffered, the risk of death more than doubled. Sex was not found to be significantly associated with survival (HR: 4.195, p=0.0742).

**Figure 1:**
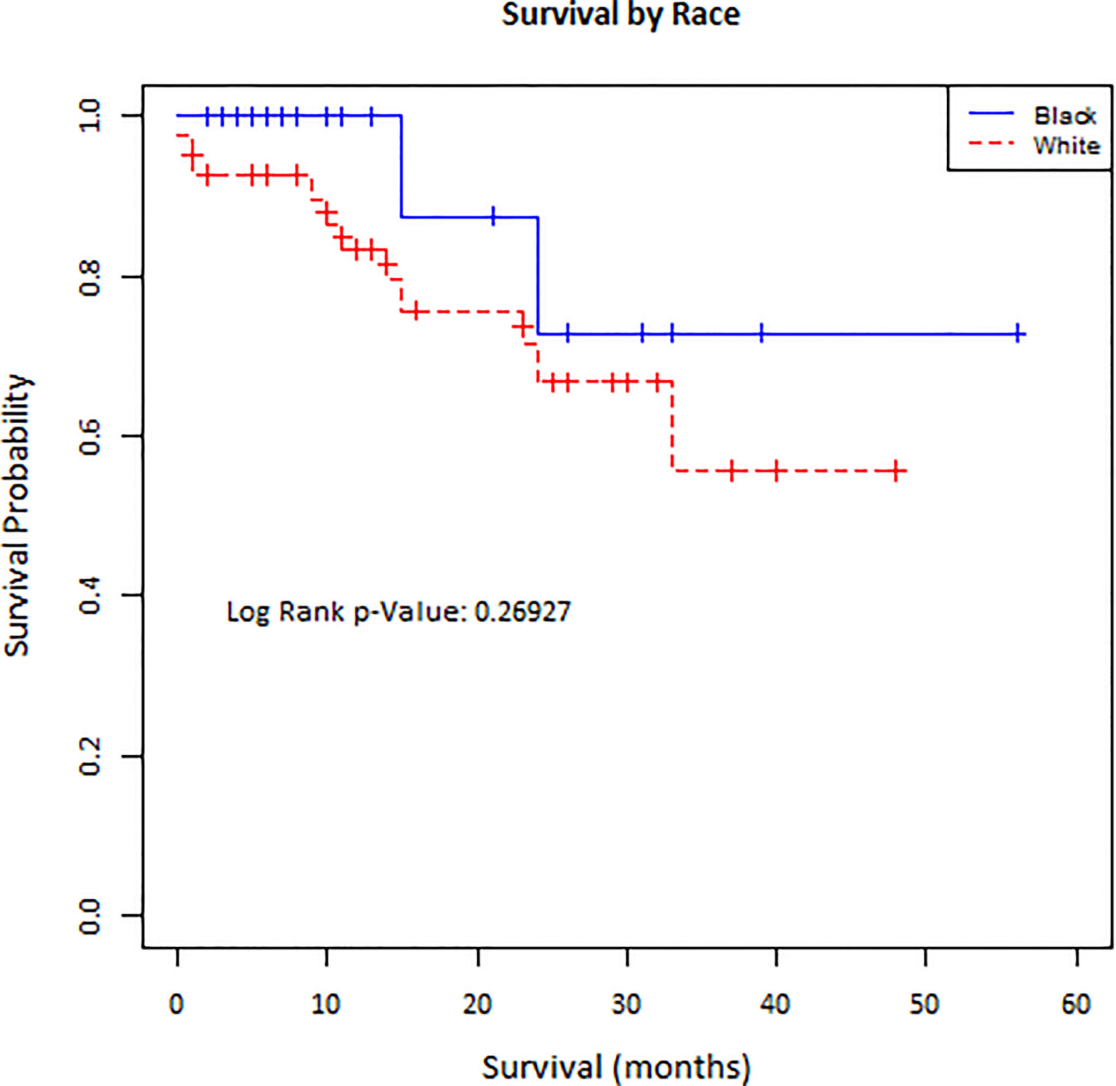
Kaplan-Meier curve of survival for Black and White patients undergoing CRS and HIPEC.

Survival analysis was completed to compare overall survival between Black and White patients with peritoneal carcinomatosis treated with CRS and HIPEC (**Figure 1**). There was no statistically significant difference in the distribution of survival time among Black and White patients (p=0.2693).

### Power Analysis

A power analysis for sample size was performed using a two-sided Log Rank Test to ensure the results were not underpowered. Using an overall sample size of 61 subjects (20 Black patients and 41 White patients) a power of 80.5% at a 5% significance level was achieved to detect a hazard ratio of 2.3 (corresponding to a moderate effect size of 0.65, under exponentiality assumptions for the survival functions) between the comparison groups. Two assumptions were made including 4.6 years of follow-up based on the maximum follow-up time of 56 months and no subjects dropping out of the study. This indicates that the study is not underpowered.

## Discussion

Health disparity research is at the forefront of cancer research in efforts to establish more equitable care across diverse patient populations. Despite low awareness among surgeons, the surgical management of cancer is fraught with disparities (1, 17, 18, 20–23). There is sparce literature examining health disparities in patients with peritoneal carcinomatosis treated with CRS and HIPEC with no literature evaluating the presence of racial disparities in this population. We present a patient population with comparable preoperative demographics and risk factors, who had similar perioperative outcomes. Despite having lower income and presumably higher financial vulnerability, Black patients in our study had similar outcomes compared to White patients. This finding contrasts the results reported by Rieser et al. who found that for colorectal cancer patients with peritoneal carcinomatosis, patients with lower socioeconomic status had longer lengths of stay, more complications, and higher rates of 90-day readmission and 30-day mortality (7). The authors argued that patients with lower socioeconomic status experience multiple disadvantages and worse overall survival compared to higher socioeconomic status patients that was not explained by individual cancer biology characteristics. Locoregional differences may influence why our results do not corroborate the findings reported by Rieser et al.

When comparing racial groups, hypertension was the only comorbidity that disproportionately affected Black patients, consistent with prevalence rates of hypertension nationally (24). Interestingly, however, there were no differences in the rates of other comorbidities, although Black patients share a higher burden of diseases such as chronic kidney disease and diabetes (25, 26). This likely reflects the underlying referral and selection patterns for patients that are relatively healthy at baseline and can withstand a complex and morbid surgery.

Previous studies have reported the association of insurance status with overall survival, but insurance was not a predictive factor in our patient population (8, 9). Overall survival was similar between Black and White patients in our study. Stratification by insurance status is the only other sociodemographic factor that has been examined for difference in overall survival with varying results. In a 2021 study of 124 patients with colorectal cancer receiving CRS and HIPEC, patients who were underinsured had worse survival than insured patients (8). However, in a smaller study of 31 patients with varying cancers undergoing CRS and HIPEC, there was no difference in survival by insurance status (9).

We also found that the occurrence of a major complication postoperatively was associated with overall survival. This agrees with results from a similar study investigating colorectal cancer patients undergoing CRS and HIPEC (7).

Notably, our study represents a diverse patient population. Nearly one third of the patients were Black. This is remarkable because other studies have reported proportions of 10% or less, or race was not reported (7, 8). In a 2019 study of the National Cancer Database characterizing the patient population undergoing cytoreductive surgery and perioperative chemotherapy (defined as receipt of HIPEC at the time of surgery or intraperitoneal chemotherapy in the perioperative period) for appendiceal cancer, only 6.60% of patients were reported as Black race, and the majority, 88.2%, were reported as White race, likely disproportionately representing the diversity of the patients with appendiceal cancer (27). There is otherwise a paucity of literature characterizing the racial distribution of patients that undergo CRS and HIPEC. This raises the larger and more concerning question as to why so few Black patients compared to White patients are receiving CRS and HIPEC, when incidence rates of some cancers treated with CRS and HIPEC are higher among Black patients (28). This also raises the question of what, if any, underlying factors may be preventing this population from potentially receiving treatment.

Our results argue that since postoperative and oncologic outcomes are similar between Black and White patients, Black patients with peritoneal carcinomatosis should be referred for and treated with CRS and HIPEC equitably. However, Byrne et al. reported that in patients with appendiceal cancer, White race and non-Hispanic ethnicity were both positive predictors for receiving CRS and HIPEC (OR: 2.00, 95% CI 1.40-2.86; OR: 1.92, 95% CI 1.21-3.05, respectively) (27). Given that CRS and HIPEC are complex and highly specialized procedures primarily conducted at tertiary care centers, the level of specialization itself may potentially be contributing to lack of access to care. Previous research has shown that hospital factors are influential in racial health disparities for cancer surgery (1, 21). When compared to White patients, Black patients with colorectal cancer were less likely to be referred to high-volume hospitals for the treatment of their cancer (29). However, racial disparities were erased when patients received care for colorectal cancer in the setting of an equal access healthcare system (22).

We do acknowledge the multiple limitations of our study. First, these findings are from a small sample over a four-year timeframe, representative of a single institution’s patient population. Therefore, these results may not be applicable to the entire patient population that undergoes this procedure and may reflect the high-quality equitable care delivered at our institution. Although our power analysis indicates that our results are not underpowered, we acknowledge that the sample size is small and further investigation with larger sample sizes is warranted. Second, given limited sample sizes within each cancer type, all patients treated with CRS and HIPEC were grouped together without stratifying for different cancer types. Doing so may potentially neglect the underlying and unique biologic and physiologic differences of each cancer that influence survival. However, this lack of stratification holds validity for viewing CRS and HIPEC as a treatment modality for peritoneal carcinomatosis regardless of the cancer type. Lastly, although racial disparities in referral for or access to CRS and HIPEC are important to assess, such an analysis extends beyond the scope of this current study, which demonstrates that once this treatment modality has been accessed, outcomes are similar regardless of race.

Despite these limitations, these results are encouraging, yet necessitate the need for further investigation. Specifically, further study must be undertaken to investigate any possible racial/ethnic disparities in larger, more nationally diverse and representative patient populations, in hopes of confirming our findings. Additionally, as previously mentioned, there is little data characterizing the patient population that is actually receiving CRS and HIPEC for peritoneal carcinomatosis. This presents an opportunity to better examine which populations are undergoing CRS and HIPEC, and to identify what factors and disparities are present that limit access to a possible cure for cancer.

In conclusion, advanced cancer states perpetuate health disparities, especially with respect to race. We hypothesized that the advanced cancer state of peritoneal carcinomatosis would demonstrate such racial disparities. Our results, however, contradicted this, and demonstrated that regardless of a patients’ race, outcomes are similar after CRS and HIPEC, despite differences in socioeconomic status and comorbidities. Therefore, aggressive surgical management of peritoneal carcinomatosis promotes equitable outcomes across diverse patient populations and more efforts should be taken to investigate disparities in this patient population.

## Data Availability Statement

The raw data supporting the conclusions of this article will be made available by the authors, without undue reservation.

## Ethics Statement

The studies involving human participants were reviewed and approved by Virginia Commonwealth University IRB. Written informed consent for participation was not required for this study in accordance with the national legislation and the institutional requirements.

## Author Contributions

DF designed the project, acquired, analyzed and interpreted data, and wrote the manuscript. XD and DB analyzed and interpreted data. VV, AR, and KH interpreted data and revised the manuscript. LF and JT designed the project and revised the manuscript. All authors contributed to the manuscript, approved the submitted version, and are accountable for the content of the work.

## Funding

Services and products in support of the research project were generated by the VCU Massey Cancer Center Biostatistics Shared Resource, supported, in part, with funding from NIH-NCI Cancer Center Support Grant P30 CA016059. The authors are additionally supported by the National Human Genome Research Institute (T32 HG008958 to AR, KH) and National Cancer Institute (R01 CA242003 to JGT, U54 CA233444 to JGT, U54 CA233444-03S1 to AR and JT, and T32 CA093423-13 to DCF) of the National Institutes of Health and the Joseph and Ann Matella Fund for Pancreatic Cancer Research (JT).

## Conflict of Interest

The authors declare that the research was conducted in the absence of any commercial or financial relationships that could be construed as a potential conflict of interest.

## Publisher’s Note

All claims expressed in this article are solely those of the authors and do not necessarily represent those of their affiliated organizations, or those of the publisher, the editors and the reviewers. Any product that may be evaluated in this article, or claim that may be made by its manufacturer, is not guaranteed or endorsed by the publisher.
