# Supplementary material for: Racial Disparities in Cytoreductive Surgery and Hyperthermic Intraperitoneal Chemotherapy: Does Aggressive Surgical Treatment Overcome Cancer Health Inequities?
Source: Front Oncol. 2022 Jun 8;12:899488. doi: 10.3389/fonc.2022.899488 (PMC9213675; doi:10.3389/fonc.2022.899488)
Supplement: Supplementary file 1 [file Table_1.docx]

**Supplemental Table 1.** Postoperative complications within 30 days of surgery by race.

|  | **Black (n=20)** | **White (n=41)** | ***p*-value** |
| --- | --- | --- | --- |
| Myocardial infarction | 0 (0.0%) | 1 (2.4%) | 1.0000 |
| Atrial fibrillation | 0 (0.0%) | 2 (4.9%) | 1.0000 |
| Pneumonia | 2 (10.0%) | 5 (12.2%) | 1.0000 |
| Pulmonary embolus | 1 (5.0%) | 1 (2.4%) | 1.0000 |
| Prolonged intubation (>48 hr) | 3 (15.0%) | 0 (0.0%) | 0.0317 |
| Reintubation | 0 (0.0%) | 5 (12.2%) | 0.1620 |
| Urinary tract infection | 2 (10.0%) | 6 (14.6%) | 1.0000 |
| Acute kidney injury | 1 (5.0%) | 9 (22.0%) | 0.1440 |
| Renal failure | 0 (0.0%) | 1 (2.4%) | 1.0000 |
| Blood transfusion | 6 (30.0%) | 17 (41.5%) | 0.3858 |
| Ileus | 8 (40.0%) | 9 (22.0%) | 0.1400 |
| Intra-abdominal infection | 1 (5.0%) | 4 (9.8%) | 1.0000 |
| Anastomotic leak | 1 (5.0%) | 1 (2.4%) | 1.0000 |
| Enterocutaneous fistula | 0 (0.0%) | 1 (2.4%) | 1.0000 |
| Gastrointestinal bleed | 1 (5.0%) | 1 (2.4%) | 1.0000 |
| Intra-abdominal hemorrhage | 1 (5.0%) | 1 (2.4%) | 1.0000 |
| Fascial dehiscence | 0 (0.0%) | 2 (4.9%) | 1.0000 |
| Abdominal compartment syndrome | 0 (0.0%) | 1 (2.4%) | 1.0000 |
| Surgical site infection | 0 (0.0%) | 3 (7.3%) | 0.5443 |
| Bacteremia | 0 (0.0%) | 1 (2.4%) | 1.0000 |
| Sepsis | 0 (0.0%) | 4 (9.8%) | 0.2929 |
| Procedural intervention | 1 (5.0%) | 1 (2.4%) | 1.0000 |
| Reoperation | 1 (5.0%) | 3 (7.3%) | 1.0000 |
